# *Cyclamen persicum* Bulb Extract Modulates NF-κB, Oxidative Stress, and Apoptotic Pathways in Triple-Negative Breast Cancer

**DOI:** 10.3390/ph19030388

**Published:** 2026-02-28

**Authors:** Aya Sharara, Rola Abdallah, Adnan Badran, Rami A. Abdel-Rahem, Mayyas Al-Remawi, Serine Baydoun, Marc Maresca, Elias Baydoun

**Affiliations:** 1 Department of Biology, American University of Beirut, Beirut P.O. Box 110236, Lebanon; aya.sharara@st.ul.edu.lb (A.S.); rha62@mail.aub.edu (R.A.); 2 Department of Biology, University of Jordan, Amman P.O. Box 11942, Jordan; adnan.badran@ju.edu.jo; 3 Department of Chemistry, University of Petra, Amman P.O. Box 961343, Jordan; rabdelrahem@uop.edu.jo; 4 Department of Pharmaceutics and Pharmaceutical Technology, University of Petra, Amman P.O. Box 961343, Jordan; malremawi@uop.edu.jo; 5 Breast Imaging Section, Imaging Institute, Cleveland Clinic Foundation, Cleveland, OH 44195, USA; baydous@ccf.org; 6 Aix Marseille Univ, CNRS, Centrale Marseille, iSM2, 13013 Marseille, France

**Keywords:** triple negative breast cancer, *Cyclamen persicum*, apoptosis, NF-κB

## Abstract

**Background/Objectives**: Triple-negative breast cancer (TNBC) is an aggressive breast cancer subtype associated with poor prognosis and limited targeted therapeutic options. Natural products, rich in bioactive phytochemicals, represent a potential source of novel anticancer agents. This study examined the phytochemical profile and anticancer activity of an ethanolic bulb extract of *Cyclamen persicum* (CPE), with a primary focus on TNBC. **Methods**: The phytochemical composition of CPE was analyzed by liquid chromatography–mass spectrometry (LC–MS). Antioxidant activity was evaluated using DPPH radical scavenging assay. The anticancer effects of CPE were assessed mainly in MDA-MB-231 TNBC cells using MTT cell viability assays, Ki-67 immunoblotting, Western blot analysis of signaling proteins, wound healing migration assays, Matrigel invasion assays, adhesion assays and cell–cell aggregation assays. Antiproliferative activity was also examined in 22RV1 (prostate), Capan-2 (pancreatic), and HCT116 (intestinal) cancer cell lines using MTT assays. **Results**: LC–MS analysis indicated that the extract contains multiple polyphenolic and organic acid constituents commonly associated with bioactivity. Consistent with this profile, CPE demonstrated strong antioxidant activity. In MDA-MB-231 cells, CPE significantly reduced cell viability and proliferation, accompanied by decreased Ki-67 expression. Treatment was associated with modulation of proteins involved in proliferative and survival signaling, induction of apoptosis-related markers, and reduced migratory and invasive capacities. CPE also promoted cell–cell homotypic aggregation, suggesting a shift toward a less aggressive phenotype. These effects were associated with reduced phosphorylation of p65, indicating possible modulation of NF-κB signaling. Additionally, CPE decreased proliferation in 22RV1, Capan-2, and HCT116 cancer cell lines. **Conclusions**: Collectively, these findings indicate that *C. persicum* bulb extract exerts multimodal anticancer effects in vitro, particularly in TNBC cells, and highlights its potential as a source of bioactive compounds warranting further mechanistic and translational investigation.

## 1. Introduction

Cancer is one of the top causes of mortality globally, with a projected 10 million deaths from the disease in 2020 [1]. Breast cancer ranks fifth among cancer-related deaths and represents the most common type of newly diagnosed cancer [2]. Several measures, including screening programs and general preventive practices, are crucial for breast cancer early detection and for potentially reducing its incidence [3]. Currently, the Breast Health Global Initiative is responsible for developing appropriate guidelines and strategies to ensure effective global breast cancer control [4]. Despite substantial progress in treatment protocols and modalities, surgery, chemotherapy, and radiation remain the primary therapeutic approaches for most breast cancer types [5]. Targeted and hormonal therapies can be used for carcinomas expressing hormone receptors (HRs) such as estrogen receptors (ER), progesterone receptors (PR), and human epidermal growth factor receptor 2 (HER2) [6]. However, TNBC, which lacks ER, PR, and HER2 expressions, represents among the most aggressive subtypes of breast cancer. It is associated with poor prognosis, high recurrence rates, and limited targeted treatment options, underscoring the need for alternative therapies, including plant-derived agents [7,8].

Herbal medicine is widely used and increasingly popular. It is believed to serve not only for treating diseases but also for promoting health and preventing illness [9]. These herbal cures and natural items are often regarded as better substitutes, with fewer side effects than conventional drugs [10]. Moreover, plants have long been utilized in cancer therapy and have provided the foundation for several modern anticancer agents [11]. Although several flavonoid- and polyphenol-rich plant extracts have been reported to suppress proliferation and induce apoptosis in cancer cells [12,13], the anticancer activity of individual medicinal plants remains highly context-dependent. In particular, the effects of *Cyclamen persicum* on TNBC, especially with respect to tumor aggressiveness and metastatic behavior, remain poorly characterized.

*Cyclamen persicum* (CP) is one of the most decorative pot plant found in various countries [14]. It belongs to the family Primulaceae and the genus *Cyclamen*, which comprises 24 species of perennial flowering plants [15]. It grows in temperate Mediterranean climates and is native to Europe and the Mediterranean Basin, extending eastward to the Caucasus and Iran, with one species (*C. somalense*) native to Somalia [15]. The therapeutic benefits of *Cyclamen* have long been recognized by the Romans, Greeks, and Egyptians [16]. Traditionally, *C. persicum* was used to treat headaches, muscle spasms, and toothaches [16]. Its tubers were also employed to manage arthritic and rheumatic disorders [16]. Today, *C. persicum* is widely cultivated as both an ornamental and medicinal plant due to its diverse bioactive compounds [16]. *C. persicum* has been reported to be rich in polyphenols, flavonoids, terpenoids, and saponins [17]. These bioactive molecules confer antioxidants, antibacterial, anti-inflammatory, and anticancer properties to the plant [18]. *C. persicum* extracts have been reported to inhibit proliferation in HeLa, H1299, MCF-7, and PC-3 cells [19]. However, unlike previous studies that primarily examined general cytotoxicity or non-TNBC models, the present work provides a TNBC-focused, phenotype-oriented evaluation of *Cyclamen persicum* bulb extract, with particular emphasis on aggressive behaviors relevant to metastasis.

In this study, we aim to analyze the phytochemical composition of an ethanolic bulb extract of *Cyclamen persicum* and evaluate its effects on the malignant phenotype of the TNBC cell line MDA-MB-231, together with its antiproliferative activity in additional cancer cell lines, including 22RV1 (prostate), Capan-2 (pancreatic), and HCT116 (colon). This work specifically focuses on the bulb, a plant organ that has been minimally investigated despite its traditional medicinal relevance. This study was designed to evaluate CPE’s antioxidant potential and its impact on key malignant behaviors of TNBC cells, including proliferation, migration, invasion, apoptosis, and cell–cell aggregation, while investigating possible modulation of NF-κB–related signaling pathways.

## 2. Results

### 2.1. Phytochemical Profiling of C. persicum via LC–MS

It was identified that *C. persicum* contains many primary and secondary metabolites and bioactive molecules [20]. Based on the literature, *C. persicum* is rich in Rutin, Caffeic acid, Ferulic acid, Verbascosid and Quercetin [18,21]. In this study we studied the presence and absence of primary and secondary metabolites in CPE. As shown in Table 1, CPE contains tannins, phenols, flavonoids, terpenoids, anthraquinones, resins, saponins, and reducing sugars, whereas quinones, anthocyanins, steroids, and cardiac glycosides were not detected.

### 2.2. Analysis of C. persicum Bulb Extract by LC–MS

**Figure 1:**
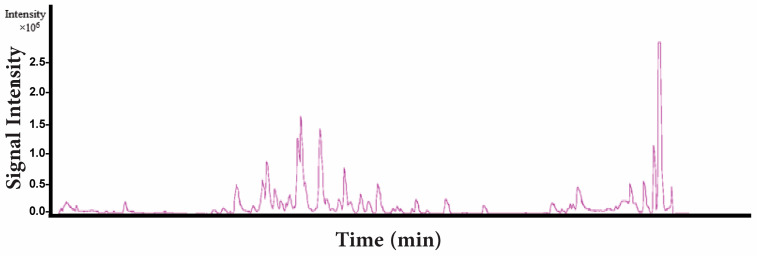
Total ion chromatogram of CPE obtained using LC–MS.

**Figure 2:**
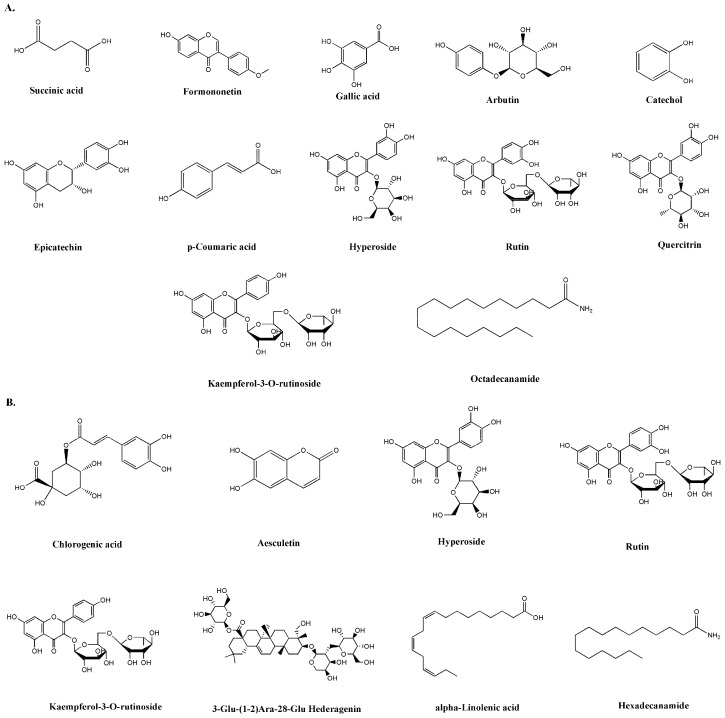
Chemical structures of the major metabolites identified in the CP bulb extract by LC–MS analysis, grouped according to ionization mode: (**A**) negative ion mode and (**B**) positive ion mode.

The *Cyclamen persicum* extract was analyzed using liquid chromatography coupled with mass spectrometry (LC–MS). The chromatographic profile revealed a rich composition of bioactive metabolites (Figure 1). High levels of epicatechin, quercitrin, and kaempferol-3-O-rutinoside were detected, along with substantial amounts of gallic acid, catechol, and succinic acid. These compounds are known for their strong antioxidant and anti-proliferative properties [22,23]. In addition, formononetin and octadecaneamide were identified in the bulb extract, further highlighting the chemical diversity of CP (Table 2). Figure 2 shows the chemical structures of the major metabolites identified in the CP bulb extract by LC–MS analysis, including flavonoids, phenolic acids, an isoflavone, and a fatty acid amide. The structural diversity of the extract, including hydroxylated aromatic rings and glycosidic moieties, may contribute to its diverse in vitro biological activities.

### 2.3. CPE Significantly Suppresses the Proliferation of MDA-MB-231 Breast Cancer Cells

**Figure 3:**
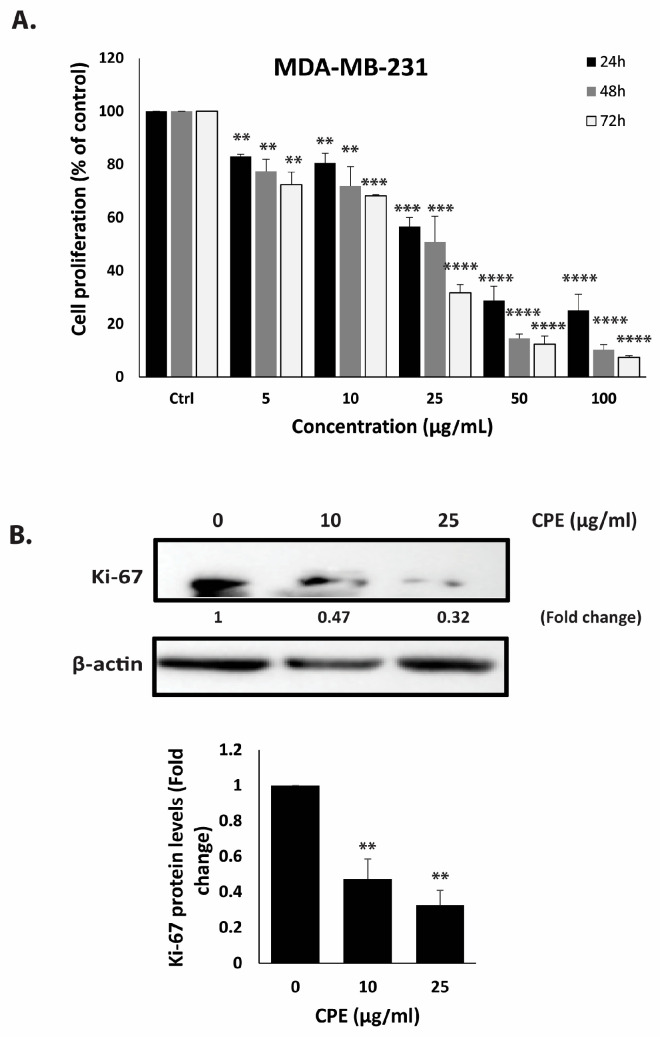
MDA-MB-231 cell growth is inhibited by CPE. (**A**) The MTT test was used to measure cell proliferation after cells were exposed to the specified doses of CPE for 24, 48, and 72 h. The mean ± SEM of three separate, triplicate trials (*n* = 3) was used to display the data. (**B**) Western blotting was used to measure the expression of Ki-67 protein after cells were treated with either vehicle control or CPE (10 or 25 µg/mL). The loading control was β-actin. The mean ± SEM of three separate studies (*n* = 3) is represented by Western blot quantification. ** *p* < 0.01, *** *p* < 0.001, and **** *p* < 0.0001.

It has been reported that several bioactive compounds identified in *Cyclamen persicum*, including apigenin, naringenin, carvacrol, thymoquinone, thymol, and rosmarinic acid, possess potent anti-breast cancer properties and are capable of reducing the malignant phenotype in various BC cell lines [5]. Based on these findings, a TNBC cell line, MDA-MB-231, was selected to investigate the potential anticancer effects of *C. persicum* bulb ethanolic extract. To assess the cytotoxic activity of CPE, MDA-MB-231 cells were treated with different concentrations (0, 5, 10, 25, 50, and 100 μg/mL) for 24, 48, and 72 h. The results demonstrated that CPE significantly decreased cell proliferation in a concentration- and time-dependent manner (Figure 3). Notably, after 72 h of treatment, the proliferation of MDA-MB-231 cells treated with 5, 10, 25, 50, and 100 μg/mL of CPE was 72.3 ± 4.8%, 68.1 ± 0.4%, 31.6 ± 3.06%, 12.3 ± 3.06%, and 7.4 ± 0.6%, respectively, relative to untreated control cells (Figure 3A). For comparison, MDA-MB-231 cells were also treated with ethanolic extracts of *C. persicum* leaves under the same conditions. The leaf extract showed minimal antiproliferative activity (Figure S1), confirming that the bulb is the primary source of bioactive compounds responsible for the observed anticancer effects.

To validate the anti-proliferative effects of CPE, protein lysates from treated MDA-MB-231 cells were subjected to immunoblotting using an antibody against Ki67, a widely recognized marker for cell proliferation and a prognostic indicator in multiple cancers. TNBC is characterized by elevated Ki67 expression, which correlates with aggressive tumor behavior and poor clinical outcomes [8]. As shown in Figure 3B, treatment with 10 and 25 μg/mL CPE reduced Ki67 protein levels to 0.47- and 0.32-fold, respectively, relative to vehicle-treated controls, demonstrating a concentration-dependent decrease. Together, the results presented in Figure 3A,B confirm that CPE effectively suppresses the proliferation of MDA-MB-231 cells.

**Figure 4:**
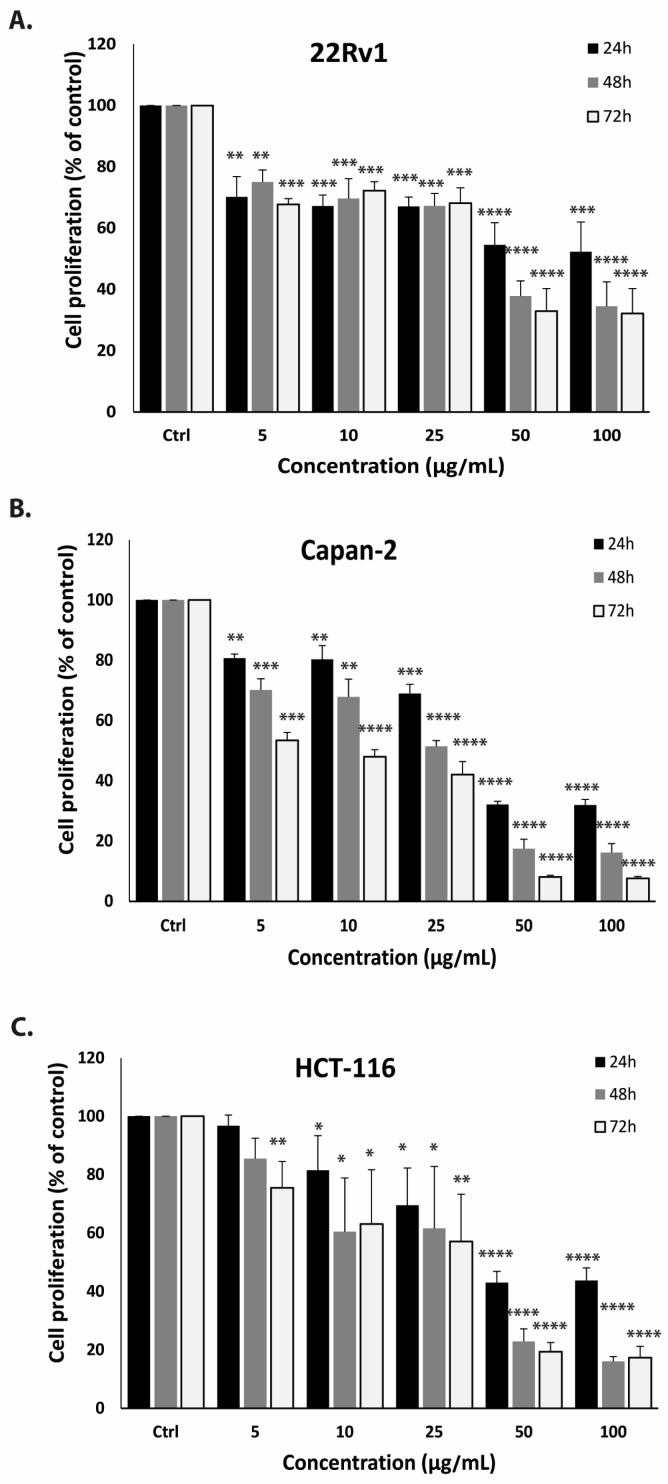
CPE reduces the proliferation of multiple cancer cell lines. The effect of CPE on cell proliferation was evaluated using the MTT assay in (**A**) 22RV1, (**B**) Capan-2, and (**C**) HCT116 cells following treatment with indicated concentrations. The data are reported as a percentage in relation to untreated control cells and are displayed as the mean ± SEM of three separate experiments conducted in triplicate. * *p* < 0.05, ** *p* < 0.01, *** *p* < 0.001, and **** *p* < 0.0001.

CPE was found to inhibit cell proliferation not only in MDA-MB-231 cells but also across multiple cancer cell lines. The cytotoxic effects of CPE were evaluated using the MTT assay in 22RV1 (prostate cancer), Capan-2 (pancreatic cancer), and HCT116 (colon cancer) cell lines. As shown in Figure 4, treatment with CPE for 72 h led to a concentration-dependent reduction in cell proliferation. In 22RV1 cells (Figure 4A), cell proliferation decreased to 67.76 ± 1.88%, 72.22 ± 2.95%, 68.16 ± 4.97%, 32.90 ± 7.38%, and 32.16 ± 8.10% at 5, 10, 25, 50, and 100 μg/mL, respectively, relative to untreated controls. Similarly, in Capan-2 cells (Figure 4B), cell proliferation was reduced to 53.43 ± 2.66%, 47.99 ± 2.31%, 42.05 ± 4.32%, 8.07 ± 0.60%, and 7.60 ± 0.61%, and in HCT116 cells (Figure 4C), cell proliferation decreased to 75.45 ± 9.01%, 63.08 ± 18.56%, 57.10 ± 16.16%, 19.38 ± 3.17%, and 17.83 ± 6.74% at the same concentrations. These results confirm that CPE exerts antiproliferative effects in vitro across multiple cancer cell lines.

### 2.4. CPE Has High Antioxidant Activity and Induces ROS Production in MDA-MB-231 Cells

**Figure 5:**
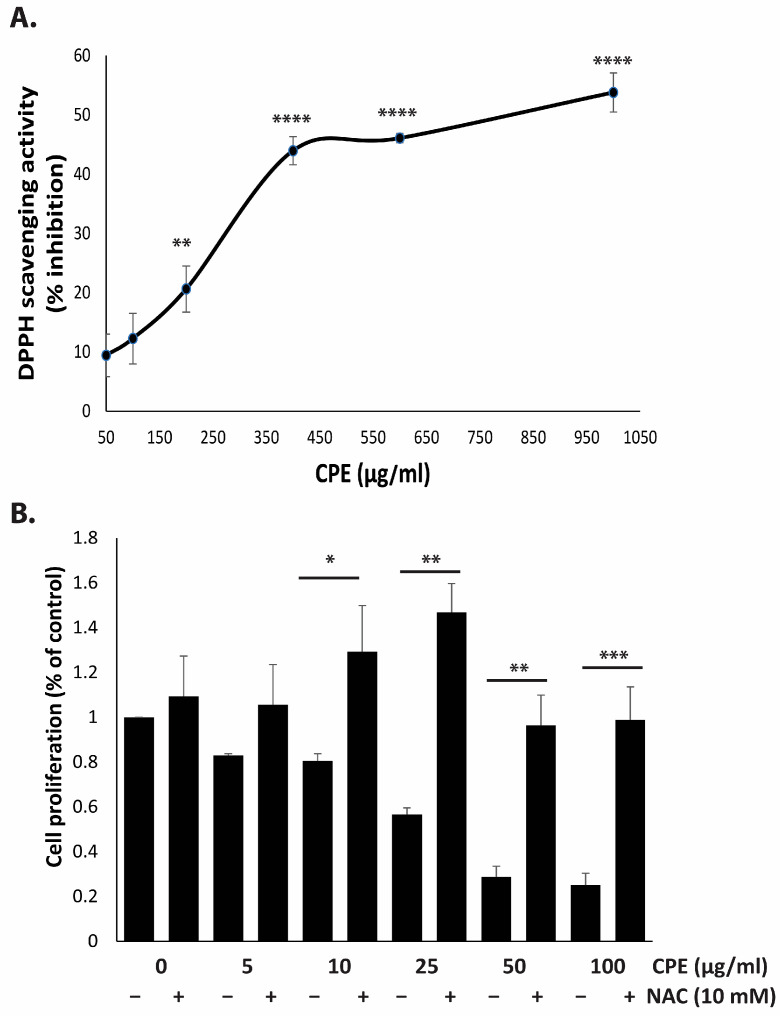
CPE demonstrates strong antioxidant activity and modulates ROS in MDA-MB-231 cells. (**A**) The free-radical scavenging activity of CPE was measured using the DPPH assay at the indicated concentrations. Data are presented as mean ± SEM of three independent experiments. (**B**) Cells were pre-treated with NAC (10 mM) for 30 min, followed by exposure to CPE (5, 10, 25, 50, or 100 μg/mL) for 24 h. Cell proliferation was determined using the MTT assay. Data represent mean ± SEM of three independent experiments. * *p* < 0.05, ** *p* < 0.01, *** *p* < 0.001, and **** *p* < 0.0001.

Reactive oxygen species (ROS) play a dual role in cellular physiology, participating in normal signaling processes but also contributing to pathological conditions, including cancer. Both ROS and antioxidants have been shown to exhibit pro- or anti-carcinogenic effects, depending on their levels and context [24,25]. In this study, CPE demonstrated substantial reducing power, with a free radical scavenging activity of 46.09 ± 0.17% at 600 µg/mL. Qualitative analyses revealed that the extract is rich in phenolic and flavonoid compounds, consistent with LC–MS profiling. The DPPH assay confirmed that CPE exhibits concentration-dependent antioxidant activity, suggesting that its phytochemicals may contribute to ROS modulation (Figure 5A).

To investigate whether the anti-proliferative effects of CPE are ROS-dependent, MDA-MB-231 cells were pre-treated with NAC, a ROS scavenger, prior to CPE exposure. Cell proliferation assay after 24 h demonstrated that NAC significantly reduced CPE-induced cytotoxicity (Figure 5B). Specifically, treatment with 10 µg/mL CPE for 24 h decreased cell proliferation to 80.5 ± 3.1%, which increased to 129.29 ± 20.5% with NAC pre-treatment. Similarly, treatment with 25 µg/mL CPE reduced proliferation to 56.6 ± 2.9%, which was restored to 146.9 ± 12.6% following NAC pre-treatment. These results indicate that the anti-proliferative effects of CPE on TNBC cells are ROS-dependent, likely mediated by its high content of phenolic and flavonoid compounds.

### 2.5. CPE Induces Intrinsic Apoptosis in MDA-MB-231 Cells

**Figure 6:**
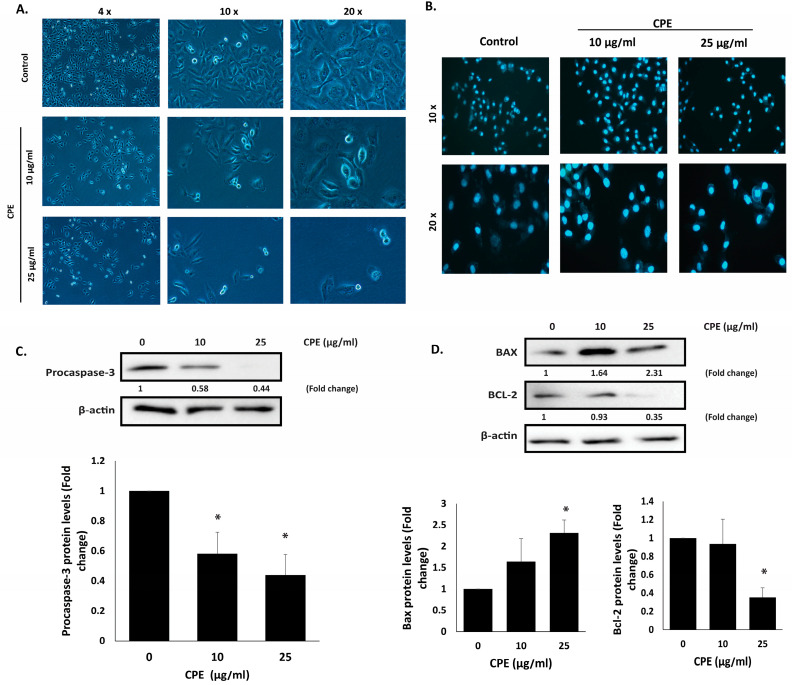
CPE triggers intrinsic apoptosis in MDA-MB-231 cells. (**A**) Cells were treated with the indicated concentrations of CPE for 24 h, and morphological changes were examined using microscopy. (**B**) Nuclear condensation and fragmentation were assessed in DAPI-stained cells following 24 h treatment with CPE. (**C**,**D**) Western blot analysis was performed to determine the protein levels of pro-caspase-3, BCL-2, and BAX. Data are presented as fold change relative to vehicle-treated controls and represent the mean ± SEM of three independent experiments. * *p* < 0.05.

The morphological changes in MDA-MB-231 cells following 24 h treatment with CPE were examined using an inverted phase-contrast microscope. CPE-treated cells displayed typical apoptotic features, including nuclear abnormalities, membrane blebbing, and cell shrinkage, along with a concentration-dependent decrease in the number of viable cells per field (Figure 6A). DAPI staining further confirmed these observations, showing chromatin condensation, nuclear fragmentation, and the formation of apoptotic bodies, all hallmarks of apoptotic cell death (Figure 6B).

Caspase-3, a central executioner of apoptosis, is activated via cleavage of its inactive precursor, pro-caspase-3 [26]. To investigate the mechanism of CPE-induced apoptosis, pro-caspase-3 protein levels were assessed. Treatment with 25 μg/mL CPE caused a significant reduction in pro-caspase-3 expression (0.44 ± 0.13-fold), indicating enhanced cleavage and activation of caspase-3, thereby initiating the intrinsic apoptotic pathway (Figure 6C).

Additionally, The balance between anti-apoptotic BCL-2 and pro-apoptotic BAX proteins is critical for regulating mitochondrial-mediated apoptosis and contributes to chemoresistance in cancers, including TNBC [27]. In this study, CPE treatment (25 μg/mL) significantly decreased BCL-2 levels (0.35 ± 0.10-fold) while increasing BAX expression (2.31 ± 0.30-fold), confirming that CPE induces apoptosis through modulation of the intrinsic mitochondrial pathway (Figure 6D).

### 2.6. CPE Suppresses the Migratory and Invasive Behavior of MDA-MB-231 Cells

**Figure 7:**
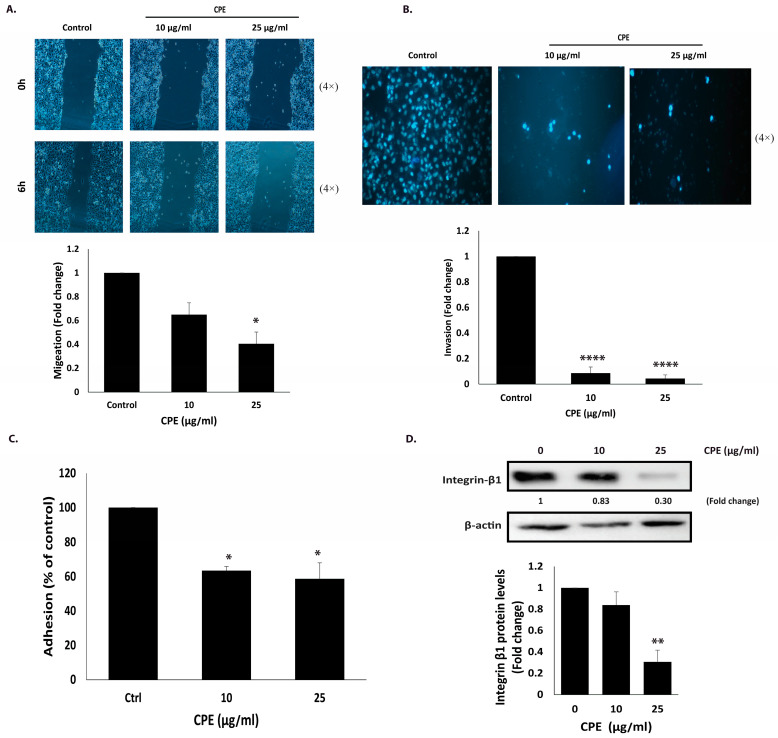
CPE inhibits migration, invasion, adhesion, and integrin β1 expression in MDA-MB-231 cells. (**A**) Migration was assessed using a scratch/wound-healing assay. Images were captured at ×4 magnification. Data are fold change relative to vehicle-treated controls and represent mean ± SEM of three independent experiments (*n* = 3). (**B**) Invasion was evaluated using Boyden chamber transwell inserts pre-coated with Matrigel. Invading cells were stained with DAPI and imaged at ×4 magnification. Data are fold change relative to control and represent mean ± SEM of three independent experiments (*n* = 3). (**C**) Adhesion was measured by seeding CPE-treated cells onto collagen-coated wells for 1 h and quantified by MTT assay. Data are expressed as a percentage of vehicle-treated controls and represent mean ± SEM of three independent experiments (*n* = 3). (**D**) Integrin β1 expression was analyzed by Western blotting after 24 h of CPE treatment. Data are fold change relative to controls and represent mean ± SEM of three independent experiments (*n* = 3). * *p* < 0.05, ** *p* < 0.01, and **** *p* < 0.0001.

Cell migration is critical for normal physiological processes such as wound healing, tissue development, and immune responses. However, uncontrolled migration beyond the primary tumor site contributes to early cancer metastasis. To examine the effect of CPE on MDA-MB-231 cell motility, a wound healing scratch assay was performed. As shown in Figure 7A, CPE treatment inhibited cell migration, reducing the ability of cells to close the scratch. Specifically, 6 h after creating the wound, migration was decreased to 0.65 ± 0.09-fold and 0.40 ± 0.09-fold in cells treated with 10 and 25 μg/mL CPE, respectively, compared to untreated controls.

Cell invasion, a key step in early metastasis, allows cancer cells to penetrate surrounding tissues and spread from the primary tumor. To evaluate the effect of CPE on invasive potential, MDA-MB-231 cells were subjected to Matrigel-coated trans-well assays in the presence or absence of CPE (10 and 25 μg/mL). CPE treatment markedly reduced the number of invading cells reaching the lower chamber, with invasion levels of 0.086 ± 0.047-fold and 0.043 ± 0.028-fold at 10 and 25 μg/mL, respectively, relative to controls (Figure 7B). These results demonstrate that CPE significantly impairs both migration and invasion in MDA-MB-231 cells, indicating a strong anti-metastatic effect.

### 2.7. CPE Reduces the Adhesion of MDA-MB-231 Cells to Matrigel

Cell adhesion plays a crucial role in intercellular communication, regulation, and the structural integrity of tissues. To evaluate the effect of CPE on the adhesive properties of MDA-MB-231 cells, a collagen adhesion assay was performed. CPE treatment significantly reduced cell adhesion to collagen by 63.45 ± 2.6% and 58.6 ± 9.2% at 10 and 25 μg/mL, respectively (Figure 7C). To further validate these findings, the expression of integrin β1, a key adhesion molecule, was examined by Western blot analysis. Notably, integrin β1 expression was decreased to 0.83 ± 0.12-fold and 0.30 ± 0.10-fold following treatment with 10 μg/mL and 25 μg/mL CPE, respectively (Figure 7D), supporting the observed inhibition of cell adhesion.

### 2.8. CPE Promotes Cell–Cell Aggregation in MDA-MB-231 Cells

**Figure 8:**
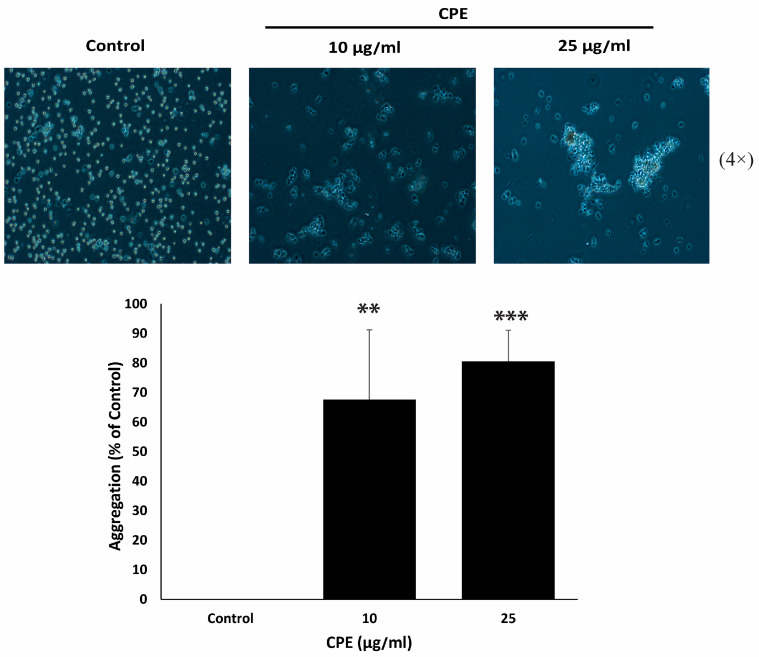
CPE promotes cell–cell homotypic aggregation in MDA-MB-231 cells. Cells were treated with the indicated concentrations of CPE and analyzed using a cell-aggregation assay. Images were captured at ×4 magnification. Percentage aggregation was calculated as: % aggregation = (1 − Nt/Nc) × 100, where Nt and Nc are the numbers of single cells in treated and control groups, respectively. Data are mean ± SEM of three independent experiments (*n* = 3). ** *p* < 0.01, and *** *p* < 0.001.

The epithelial–mesenchymal transition (EMT) is a key process associated with tumor progression and metastasis [28] in which epithelial cells acquire migratory and invasive traits while losing intercellular adhesion. Therapeutic strategies for TNBC aim to counteract EMT, restoring epithelial characteristics and promoting cell–cell adhesion. To evaluate the effect of CPE on this process, a cell-aggregation assay was performed to assess the adhesive behavior of MDA-MB-231 cells in suspension. As shown in Figure 8, CPE treatment induced a concentration-dependent increase in cell–cell aggregation compared to untreated controls, with significant enhancements of 67.6 ± 23.6% and 80.5 ± 10.5% observed 1 h after treatment with 10 μg/mL and 25 μg/mL CPE, respectively.

### 2.9. CPE Reduces p65 Phosphorylation in MDA-MB-231 Cells

**Figure 9:**
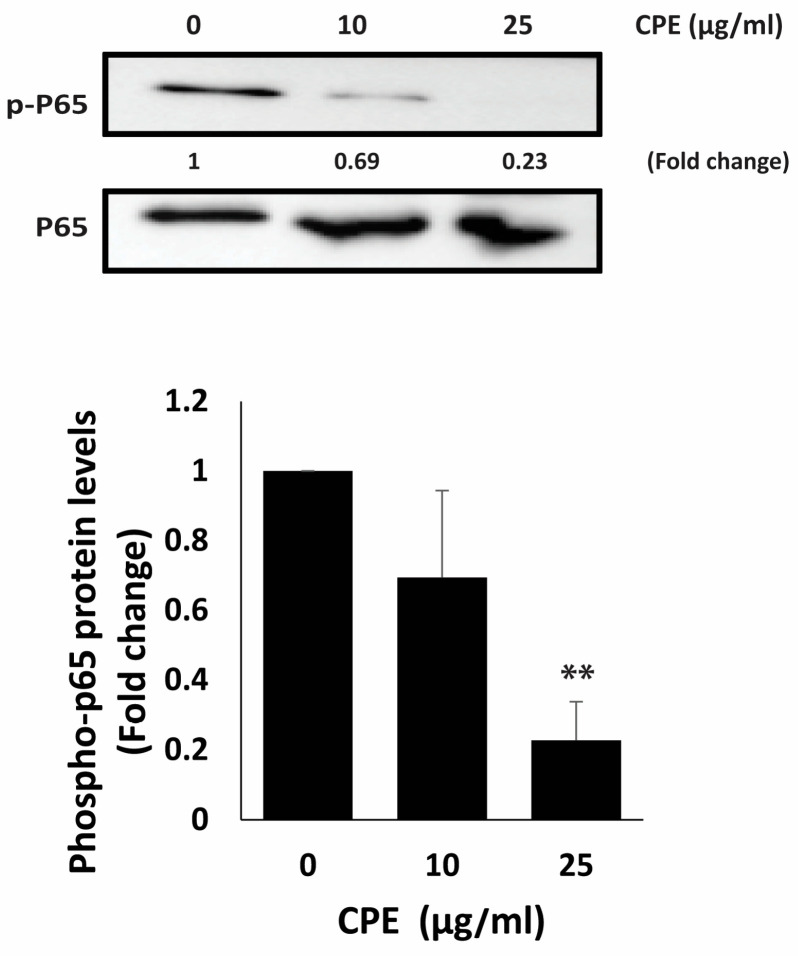
CPE reduces NF-κB phosphorylation in MDA-MB-231 cells. Cells were treated with the indicated concentrations of CPE for 24 h, and phospho-p65 levels were assessed by Western blot. Total p65 served as the loading control. Densitometric quantification is presented as fold change relative to vehicle-treated controls and represents mean ± SEM of three independent experiments (*n* = 3) ** *p* < 0.01.

To assess whether CPE modulates NF-κB signaling, we measured the phosphorylation levels of the NF-κB p65 subunit following treatment. As shown in Figure 9, CPE caused a clear dose-dependent reduction in phospho-p65 protein levels. Treatment with 10 µg/mL CPE decreased phospho-p65 to 0.69 ± 0.25-fold, while 25 µg/mL CPE produced a pronounced suppression, reducing phospho-p65 to 0.23 ± 0.11-fold of control levels. These results indicate that CPE is associated with reduced phosphorylation of p65 in MDA-MB-231 cells, suggesting a potential role in modulating NF-κB–related pro-survival and pro-metastatic signaling.

## 3. Discussion

Natural products remain an important source of anticancer agents, with several clinically approved drugs, including vincristine and paclitaxel, originating from plant-derived compounds [29]. Beyond these established examples, numerous plant metabolites have demonstrated antiproliferative, anti-migratory, anti-angiogenic, and apoptosis-modulating activities in both in vitro and in vivo models [30,31]. In this context, our phytochemical analysis indicates that *Cyclamen persicum* bulbs contain diverse classes of bioactive constituents, including phenols, flavonoids, saponins, terpenoids, tannins, anthraquinones, and resins. Despite the recognized pharmacological potential of such compound classes, limited studies have characterized the phytochemical composition or anticancer activity of *C. persicum* [32]. These considerations underscore the rationale for further systematic investigation of its biological properties.

The novelty of the present study lies in its plant-part–specific and phenotype-oriented evaluation of *Cyclamen persicum* bulb extract, rather than in proposing a new anticancer paradigm. While polyphenol-rich plant extracts have been widely reported to affect cancer cell survival, few studies have examined the biological relevance of the *C. persicum* bulb, despite its traditional medicinal use and chemical richness [13,33]. By combining phytochemical profiling with functional assays in TNBC cells, this work provides the first TNBC-focused study linking the bulb extract to suppression of proliferation, induction of apoptotic features, and inhibition of aggressive cellular behaviors such as migration and invasion. This emphasizes the importance of plant-part selection for reproducible biological effects.

LC–MS/MS profiling of *Cyclamen persicum* provided detailed characterization of the bulb extract and identified flavonoids such as epicatechin, kaempferol derivatives, and quercitrin, which may contribute to the observed antiproliferative and pro-apoptotic effects in MDA-MB-231 cells, although direct causality was not tested. Epicatechin has been reported to inhibit proliferation and promote apoptosis in TNBC cells via ROS-mediated mitochondrial pathways [34,35]. Similarly, kaempferol and its derivatives have shown anti-proliferative and anti-invasive effects, including cell-cycle arrest, modulation of MMP-9, and induction of apoptotic signaling [36,37]. Quercitrin has also been implicated in suppressing proliferation and metastasis by modulating PI3K/Akt/mTOR and Wnt/β-catenin pathways and promoting apoptosis [38,39]. Together, these phytochemicals may contribute individually or synergistically to the observed multimodal effects of CPE.

In this study, CPE significantly reduced MDA-MB-231 cell proliferation in a dose-dependent manner, accompanied by downregulation of Ki67, a marker strongly associated with tumor aggressiveness and poor prognosis in TNBC [40]. Reduced Ki67 expression reflects growth arrest and may interfere with proliferative signaling, although additional pathways could also be involved [41,42].

ROS play essential roles in cellular signaling at physiological levels, whereas excessive accumulation can disrupt redox homeostasis and promote cellular damage [43]. The balance between oxidative and antioxidant systems is therefore tightly regulated within cells [43]. Given that *Cyclamen persicum* contains polyphenolic constituents known for antioxidant activity [18], the concentration-dependent radical scavenging observed in cell-free assays was consistent with its phytochemical profile. Interestingly, pretreatment with NAC significantly attenuated the inhibitory effect of CPE on MDA-MB-231 cell proliferation, suggesting that redox modulation may contribute to the antiproliferative effects of CPE. This apparent contradiction is commonly observed with polyphenol-rich plant extracts [44,45]. Such compounds can scavenge free radicals extracellularly while modulating intracellular redox homeostasis, leading to excessive ROS production within cancer cells and triggering apoptosis [24,46,47]. However, intracellular ROS levels were not directly measured in the present study, limiting mechanistic interpretation. These findings support the concept that the anticancer potential of natural antioxidants may arise not from complete ROS elimination, but from the ability to shift intracellular ROS beyond physiological levels, selectively inducing cancer cell death [24].

CPE also modulated key regulators of the intrinsic apoptotic pathway in MDA-MB-231 cells, decreasing BCL-2 and increasing BAX levels, accompanied by reduced pro-caspase-3, consistent with pro-apoptotic signaling [48,49]. Similar effects have been reported for other plant-derived compounds: epicatechin triggers apoptosis in TNBC cells through BAX upregulation and caspase-3 activation [34], kaempferol modulates the BCL-2/BAX/caspase-3 axis to inhibit breast cancer proliferation [50], and rutin promotes apoptosis by enhancing BAX expression and caspase-3 activity [51]. While these constituents were detected in the extract, their individual contributions remain to be experimentally validated.

EMT is a critical process in cancer progression and metastasis, characterized by loss of cell–cell adhesion and altered interactions with the extracellular matrix (ECM) [52]. TNBC is particularly associated with EMT, which contributes to its aggressive phenotype [53]. In this study, CPE reduced migration and invasion, decreased cellular adhesion, and enhanced cell–cell aggregation. These effects were accompanied by a reduction in integrin levels, suggesting that CPE interferes with the ability of individual tumor cells to detach, migrate, and invade surrounding tissues. Reduced integrin expression can impair cell motility, ECM attachment, and stable adhesion to basement membranes [54]. These results suggest partial reversal of EMT in TNBC cells, which is significant given the link between EMT and drug resistance, invasion, and metastasis [55].

The NF-κB pathway, with p65 as a central subunit, regulates inflammation, immune responses, and cell survival [56]. Phosphorylation of p65 at Ser536 enhances its transcriptional activity [57]. In our study, CPE reduced the phosphorylated levels of p65, indicating possible modulation of NF-κB–associated signaling, which may partly contribute to the observed reductions in proliferation and invasiveness. Similar reductions in p65 phosphorylation have been reported for natural compounds such as curcumin and [6]-gingerol in cancer models [58,59]. These findings suggest that CPE may share a common mechanism with other natural compounds in modulating NF-κB signaling to exert anti-inflammatory and anticancer effects, but further mechanistic studies are needed to confirm this.

## 4. Materials and Methods

### 4.1. C. persicum Ethanolic Extract

The bulb *C. persicum* was collected from Baraashit, south Lebanon, at an altitude of 800 m in April 2023. The bulb was thoroughly cleaned and washed with water and dried for two weeks in the shades at room temperature followed by oven-dried at 40 °C for 24 h. After drying, the bulbs were ground into a fine powder using a grinding mill. The powder was stored in a clean plastic bag inside a desiccator to protect against moisture until use. 10 g of the fine powder were immersed in 150 mL 80% ethanol. The mixture was subjected to ultrasound treatment at 40 kHz for 1 h at under 35 °C, vacuum filtered, then processed with a rotary evaporator at 40 °C for 20 to 25 min and finally freeze-dried to obtain the extract.

### 4.2. Phytochemical Analysis

#### Preliminary Phytochemical Tests

Test for tannins: The extract 0.5 g was combined with 5 mL distilled water and subjected to a 15 min ultrasonication at 80 °C. A 0.1% ferric chloride was added following filtering and cooling. Tannins were recognized by a brownish green or blue-black coloring [60].

Test for resins: After 15 min of ultrasonication at 30 °C with 5 mL of distilled water, the 0.5 g extract was filtered. The filtrate’s turbidity indicated the presence of resins [60].

Test for saponins: After combining 0.5 g of the extract with 5 mL of distilled water, the mixture was ultrasonically heated to 80 °C for 15 min. When the solution was shook after cooling and filtering, a stable, enduring froth formed, signifying the presence of saponins [60].

Test for phenolic compounds 0.5 g of the extract was mixed with 5 mL of ethanol and ultrasonicated for 15 min at 30 °C. The mixture was filtered, and 2 mL of distilled water was added to the filtrate, followed by a few drops of 5% FeCl_3_. The appearance of a dark green color indicated the presence of phenolics [60].

Test for flavonoids: 0.2 g of the extract was mixed with 1 mL of 2% NaOH, producing a yellow-colored solution. Addition of a few drops of diluted acid rendered the solution colorless, confirming the presence of flavonoids [60].

Test for quinones: 0.5 g of the extract was mixed with 5 mL of ethanol and ultrasonicated for 15 min at 30 °C. The mixture was filtered, and 1 mL of concentrated H_2_SO_4_ was added to 1 mL of filtrate. A red color indicated the presence of quinones [60].

Test for steroids: 5 mL of ethanol and 0.5 g of the extract were combined, and the mixture was ultrasonically agitated for 15 min at 30 °C. After filtering the mixture, the filtrate was dried out by evaporating it. One milliliter of concentrated H_2_SO_4_ was added to the test tube’s side after a few milligrams of the dried extract were dissolved in 1 mL of chloroform and one ml of glacial acetic acid. A green color suggested Steroids [60].

Test for cardiac glycosides: After dissolving a few mg of the dried extract in 1 mL of glacial acetic acid with a few drops of 2% FeCl_3_, 1 mL of concentrated H_2_SO_4_ was added to the test tube’s side [60].

Test for terpenoids: 0.5 g of the extract was combined with 5 mL of chloroform and subjected to a 15 min ultra-sonication at 30 °C. After filtering the liquid, 2 mL of concentrated H_2_SO_4_ were added to the test tube’s side. A reddish-brown color confirmed terpenoids [60].

Test for anthraquinones: 0.5 g of the extract and 4 mL of benzene were combined. When a 10% ammonia solution was applied after filtering, the presence of anthraquinones was revealed by the emergence of a reddish-violet tint [60].

Test for anthocyanins: 5 mL of ethanol and 0.5 g of the extract were combined, and the mixture was ultrasonically sonicated for 15 min at 30 °C. After that, 1 mL of NaOH and 1 mL of the extract were heated to 100 °C for 5 min. Anthocyanins were recognized by a bluish-green color [60].

### 4.3. LC–MS

Five mg of *Cyclamen persicum* ethanolic bulb extract were dissolved in 50 μL of DMSO and 450 μL of methanol to create a stock solution. 250 μL was then further diluted with 1 mL methanol for use in metabolite analysis based on LC–MS. Acetonitrile, methanol, water, and formic acid were all LC–MS-grade solvents. An Elute UHPLC system connected to a Bruker Impact II ESI–QTOF mass spectrometer (Bruker Daltonik, Bremen, Germany) was used to profile metabolites. Authenticated standards helped identify compounds, allowing for accurate m/z measurement using high-resolution TOF MS and confirmation of retention durations following chromatographic separation. The following parameters were used to run the mass spectrometer using an Apollo II Ion Funnel electrospray source: capillary voltage 2500 V, nitrogen dry gas flow 8 L/min, nitrogen dry gas temperature 200 °C, and nebulizer pressure 2.0 bar. The system was able to obtain a TOF repetition rate of up to 20 kHz, a resolution of 50,000 FSR, and mass accuracy of less than 1 ppm.

A Bruker Solo 2.0 C18 UHPLC column (100 mm × 2.1 mm, 2.0 μm) kept at 40 °C with a flow rate of 0.51 mL/min was used for chromatographic separation. With a 3 μL injection volume, the mobile phases were (A) water with 0.1% methanol and (B) methanol.

Bruker MetaboScape (v5.0) and DataAnalysis (v5.1) were used for data analysis. The MetaboBase library (v3.0), an authentic standard combination, and a bespoke database of previously documented metabolites from *C. persicum* were used for compound annotation in MetaboScape. An internal R workflow utilizing the xcms (v1.54.0), CAMERA (v1.34.0), and MSnbase (v2.8.3) packages processed the raw LC–MS data after converting it to mzML format using msConvert (Prote-oWizard v3.0). The Rdisop tool (v2.13.0) was used to predict chemical formulas for annotated ions, and isotopic patterns matched theo-retical values with >99% accuracy. Bruker DataAnalysis and the ggplot2 tool in R (v3.5.2) were used to depict chromatograms and mass spectra, and both methods produced reliable findings.

### 4.4. Cell Culture

Human breast cancer (MDA-MB-231), human prostate cancer (22RV1), pancreatic ductal adenocarcinoma (Capan-2), and colon carcinoma (HCT116) cell lines (American Type Culture Collection, Manassas, VA, USA) were grown in high-glucose DMEM supplemented with 1% penicillin/streptomycin (Lonza, Basel, Switzerland) and 10% fetal bovine serum (FBS) (Sigma-Aldrich, St. Louis, MO, USA). Cells were cultured in a humidified incubator at 37 °C with 5% CO_2_.

### 4.5. Cell Proliferation Assay

MDA-MB-231, 22RV1, Capan-2, or HCT116 cells were seeded in 96-well tissue culture plates at a density of 5.0 × 10^3^ cells per well and allowed to adhere until approximately 40% confluence. Cells were then treated with increasing concentrations (5, 10, 25, 50, or 100 μg/mL) of *Cyclamen persicum* bulb extract for 24, 48, and 72 h. Cell proliferation was assessed using the 3-(4,5-dimethylthiazol-2-yl)-2,5-diphenyltetrazolium bromide (MTT) assay (Sigma-Aldrich, St. Louis, MO, USA). Cell proliferation was expressed as a percentage relative to vehicle-treated control cells (equivalent concentration of ethanol), which was considered 100%.

### 4.6. DPPH (α, α-Diphenyl-β-picrylhydrazyl) Antioxidant Activity Assay

The antioxidant activity of the ethanolic bulb extract of *Cyclamen persicum* was evaluated using the DPPH (2,2-diphenyl-1-picrylhydrazyl) radical-scavenging assay. A 0.5 mM DPPH solution made in methanol was combined with varying doses of CPE (50, 100, 200, 400, and 600 µg/mL). 0.5 mL of DPPH solution, 3 mL of methanol, and 0.5 mL of 80% ethanol made up a blank solution that was used as a reference. A spectrophotometer was used to measure the absorbance at 517 nm after the mixtures were incubated in the dark for 30 min. Using the following formula, the proportion of DPPH radical-scavenging activity was determined: Percentage of inhibition (control absorbance minus extract absorbance)/(control absorbance) × 100. Ascorbic acid served as the reference for comparison [61].

### 4.7. Microscopic Analysis of Apoptotic Morphological Changes

MDA-MB-231 cells were seeded in 6-well tissue culture plates and treated with the indicated concentrations of *Cyclamen persicum* ethanolic bulb extract or left untreated as controls. An inverted phase-contrast microscope was used to study morphological alterations indicative of apoptosis at 4×, 10×, and 20× magnifications after a 24 h period.

### 4.8. Matrigel Invasion Assay

A BD Matrigel Invasion Chamber with 8-μm pores (BD Biosciences, Bedford, MA, USA) was used to assess the invasive capacity of MDA-MB-231 cells. At a density of 1.0 × 10^3^ cells per well, cells were seeded into the top chamber and treated with either <1% ethanol as a vehicle control or the indicated quantities of *Cyclamen persicum* ethanolic bulb extract. The lower chamber was filled with DMEM containing 10% fetal bovine serum as a chemoattractant. After 24 h of incubation at 37 °C, non-invading cells on the upper surface of the insert were removed using a sterile cotton swab. Under a fluorescence microscope, cells that had moved to the lower surface of the Matrigel were counted after being fixed with 4% formaldehyde and labeled with DAPI. The results are shown as mean ± SEM, and the experiments were conducted in triplicate.

### 4.9. Wound Healing (Scratch) Assay

In 12-well plates, MDA-MB-231 cells were cultivated until they formed a continuous monolayer. A linear scratch was made across the cell layer using a 10 μL pipette tip. The wells were washed with PBS to remove any detached cells and debris, and fresh medium containing *Cyclamen persicum* ethanolic bulb extract at 10 or 25 μg/mL was added. Cells were then incubated at 37 °C. Images of the scratch were captured immediately after wounding (0 h) and at 6 h using an inverted microscope with a 4× objective. Wound closure was measured with ZEN software (v4.8, Zeiss, Oberkochen, Germany) and expressed as the mean ± SEM of the reduction in scratch width over the 6 h period.

### 4.10. Adhesion Assay

MDA-MB-231 cells were grown for 24 h with or without CPE, and then they were seeded in duplicate into 24-well collagen-coated tissue-culture dishes. Following a one-hour incubation period at 37 °C, cells that are unattached were extracted from the wells by gently washing them twice with PBS. The MTT reduction test was used to measure the adherent cell count, as previously mentioned.

### 4.11. Aggregation Assay

Using sterile 2 mM EDTA in Ca^2+^/Mg^2+^-free PBS, MDA-MB-231 cells were separated from confluent growth plates. Following its transfer to non-adherent culture plates, the resultant cell suspension was subjected to either 10 or 25 μg/mL of *Cyclamen persicum* extract. After an hour of gentle agitation (90 rpm) at 37 °C, the cells were fixed with 1% formaldehyde. After that, aggregate formation was evaluated by taking photomicrographs for analysis and visualization.

### 4.12. Analysis of Western Blots

Cells were washed twice with PBS, harvested by scraping, and lysed in 2% SDS containing 60 mM Tris buffer (pH 6.8). Cell lysates were centrifuged at 15,000× *g* for 10 min, and the protein concentration of the supernatants was determined using the Bradford protein assay kit (Bio-Rad, Hercules, CA, USA). Aliquots containing 25–30 μg of protein were separated by 10% SDS-PAGE and transferred onto polyvinylidene difluoride (PVDF) membranes (Immobilon PVDF; Bio-Rad, Hercules, CA, USA). Membranes were blocked for 1 h at room temperature with 5% nonfat dried milk in TBST (TBS with 0.05% Tween-20) and then incubated with the appropriate primary antibodies. Immunoreactive bands were visualized using the Clarity Western ECL substrate kit (Bio-Rad) according to the manufacturer’s instructions. Horseradish peroxidase-conjugated secondary antibodies were used for detection. All primary and secondary antibodies were obtained from Cell Signaling Technology (Danvers, MA, USA).

### 4.13. Statistical Analysis

Data are presented as mean ± SEM (*n* = 3 independent experiments). Comparisons between groups were performed using one-way ANOVA followed by Dunnett’s post hoc test or two-way ANOVA followed by Tukey–Kramer’s post hoc test, as appropriate. A *p*-value < 0.05 was considered statistically significant. The analyses were conducted using GraphPad Prism 9.

### 4.14. GenAI Usage

ChatGPT 4.1 was used during the preparation of this manuscript to assist with language editing, grammar correction, and improvement of clarity and readability of the text. The use of this tool did not involve data analysis, data interpretation, figure generation, or modification of scientific results. All scientific content, data interpretation, and conclusions were generated by the authors.

## 5. Conclusions

In summary, the present study provides in vitro evidence that the ethanolic bulb extract of *Cyclamen persicum* exerts anticancer effects in TNBC cells. CPE reduced MDA-MB-231 cell proliferation, migration, and invasion, promoted apoptotic markers, and enhanced homotypic cell–cell aggregation, collectively suggesting attenuation of aggressive cellular characteristics. These effects were associated with decreased phosphorylation of p65, indicating possible modulation of NF-κB–related signaling pathways implicated in TNBC progression. While these findings support the biological activity of *C. persicum* bulbs as a source of bioactive compounds, several limitations should be acknowledged. The mechanistic observations are currently restricted to a single TNBC cell line and require validation in additional TNBC models. Moreover, NF-κB involvement was inferred from p65 phosphorylation without direct assessment of transcriptional activity or downstream targets. The absence of in vivo evaluation further limits conclusions regarding biological relevance, safety, and therapeutic applicability. Overall, the present study supports further investigation into the bioactive constituents of *C. persicum* bulbs and their molecular effects in expanded cellular systems and in vivo models. Such studies will be essential to clarify mechanism, validate reproducibility across TNBC contexts, and more precisely define the biological potential of this extract.

## Figures and Tables

**Table 1:** Phytochemical analysis of ethanolic bulb extract of *Cyclamen persicum*, (+) indicates the presence and (−) indicates the absence of the secondary metabolite.

Metabolite	CPE
Anthraquinones	+
Tannins	+
Resins	+
Terpenoids	+
Flavonoids	+
Quinones	−
Anthocyanins	−
Saponins	+
Phenols	+
Steroids	−
Cardiac glycosides	−
Reducing sugar	+

**Table 2:** Compounds in the ethanolic bulb extract of *Cyclamen persicum* were identified using LC–MS’s (**A**) negative and (**B**) positive ionization modes.

(**A**) **Negative Ionization Mode**						
Number	RT [min]	m/z	Ions	Compound Name	Molecular Formula	Intensity
1	0.82	117.01871	[M-H]−	Succinic acid	C_4_H_6_O_4_	75,216
2	0.85	267.06217	[M-H]−	Formononetin	C_16_H_12_O_4_	13,127
3	1.02	125.02373	[M-H-CO_2_]-	Gallic acid	C_7_H_6_O_5_	15,772
4	1.03	169.01334	[M-H]−	Gallic acid	C_7_H_6_O_5_	49,572
5	2.04	271.06879	[M-H]−	Arbutin	C_12_H_16_O_7_	4418
6	2.17	109.02683	[M-H]−	Catechol	C_6_H_6_O_2_	38
7	5.29	289.06417	[M-H]−	Epicatechin	C_15_H_14_O_6_	11,844
8	6.18	163.03023	[M-H]−	p-Coumaric acid	C_9_H_8_O_3_	54
9	10.48	463.08615	[M-H]−	Hyperoside (Quercetin-3-O-galactoside)	C_21_H_20_O_12_	11,816
10	11.08	609.15506	[M-H]−	Rutin	C_27_H_30_O_16_	12,126
11	12.16	447.09084	[M-H]−	Quercitrin	C_21_H_20_O_11_	31,744
12	12.31	593.14838	[M-H]−	Kaempferol-3-O-rutinoside	C_27_H_30_O_15_	16,467
13	30.27	280.26308	[M-H]−	Octadecaneamid	C_18_H_35_NO	10,176
(**B**) **Positive Ionization Mode**						
14	1.31	355.09959	[M+H]+	Chlorogenic acid	C_16_H_18_O_9_	10,368.3
15	3.3	179.03363	[M+H]+	Aesculetin	C_9_H_6_O_4_	183.3
16	9.25	465.10182	[M+H]+	Hyperoside (Quercetin-3-O-galactoside)	C_21_H_20_O_12_	816.1
17	9.3	633.14262	[M+Na]+	Rutin (Quercetin-3-O-rutinoside)	C_27_H_30_O_16_	1942.0
18	9.31	611.16063	[M+H]+	Rutin (Quercetin-3-O-rutinoside)	C_27_H_30_O_16_	1168.3
19	10.96	595.16720	[M+H]+	Kaempferol-3-O-rutinoside	C_27_H_30_O_15_	2265.0
20	20	946.53552	[M+H+NH3]+	3-Glu-(1-2)Ara-28-Glu Hederagenin	C_47_H_76_O_18_	139,217.9
21	20.04	951.49268	[M+Na]+	3-Glu-(1-2)Ara-28-Glu Hederagenin	C_47_H_76_O_18_	41,808.0
22	25.21	279.23199	[M+H]+	alpha-Linolenic acid	C_18_H_30_O_2_	92,020.5
23	29.49	256.26322	[M+H]+	Hexadecanamide	C_16_H_33_NO	8,093,474.3
24	29.51	278.24560	[M+Na]+	Hexadecanamide	C_16_H_33_NO	1,942,060.9

## Data Availability

The original contributions presented in this study are included in the article/Supplementary Material. Further inquiries can be directed to the corresponding authors.

## Supplementary Materials

The following supporting information can be downloaded at: https://www.mdpi.com/article/10.3390/ph19030388/s1. Figure S1. *Cyclamen persicum* ethanolic leaf extract has no effect on the proliferation of multiple cancer cell lines.
