# Supplementary material for: Cyclamen persicum Bulb Extract Modulates NF-κB, Oxidative Stress, and Apoptotic Pathways in Triple-Negative Breast Cancer
Source: Pharmaceuticals (Basel). 2026 Feb 28;19(3):388. doi: 10.3390/ph19030388 (PMC13028942; doi:10.3390/ph19030388)
Supplement: Supplementary file 1 [file pharmaceuticals-19-00388-s001.zip › pharmaceuticals-4096887-supplementary.pdf]

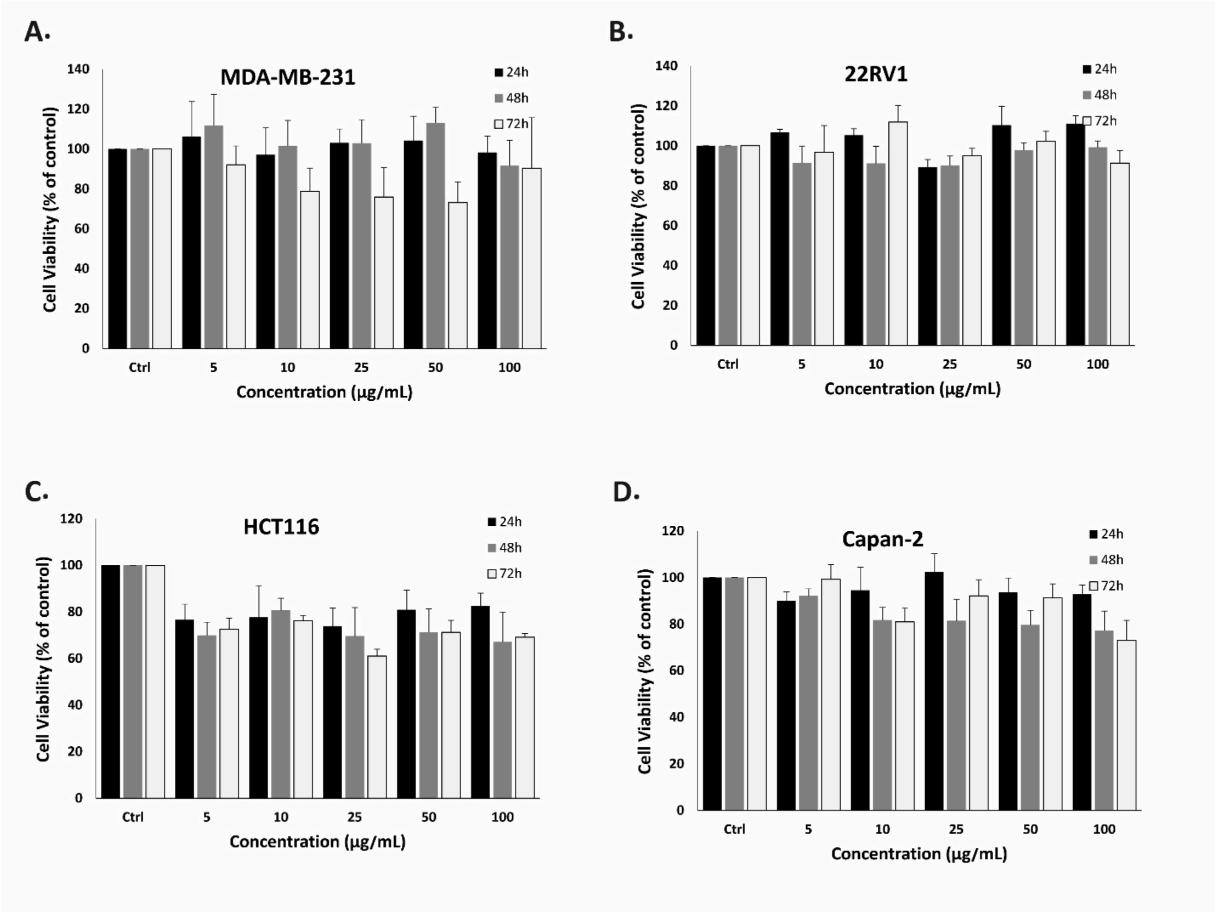

Figure S1. *Cyclamen persicum* ethanolic leaf extract has no effect on the proliferation of multiple cancer cell lines. The effect of CPE on cell proliferation was evaluated using the MTT assay in (A) MDA-MB-231, (B) 22RV1, (C) HCT116 and (D) Capan-2 cells following treatment with indicated concentrations. Data are presented as mean ± SEM of three independent experiments performed in triplicate and are expressed as a percentage relative to untreated control cells.
